# Liver Abscess: Increasing Occurrence in Premature Newborns

**Published:** 2013-04-01

**Authors:** Oktav Bosnalı, Serdar Moralıoğlu, Ayşenur Cerrah Celayir, Osman Pektaş

**Affiliations:** Department of Pediatric Surgery, Zeynep Kamil Maternity and Children’s Training and Research Hospital İstanbul, Türkiye

**Keywords:** Liver, Abscess, Neonate, Umbilical catheterization

## Abstract

Neonatal liver abscess is a very rare condition associated with high morbidity and mortality rates. There seems to be an increasing trend of this rare condition amongst the newborns admitted to neonatal intensive care units. We report a case of liver abscess in a premature newborn and briefly review the literature and discuss its management.

## INTRODUCTION

Neonatal liver abscess could be idiopathic, or secondary to umbilical infections due to umbilical vein catheterization or to sepsis which are reported to be the most common predisposing factors. Other predisposing factors include central total parenteral nutrition (TPN) catheters, necrotizing enterocolitis, umbilical and gallbladder or liver surgery, prematurity, and neutrophil defects [1,2]. Diagnosis of a liver abscess in a septic neonate requires a high index of suspicion. Ultrasonography (US) and computerized tomography (CT) with intravenous (IV) contrast are useful for diagnosing this condition. After diagnosis, correction of the predisposing factor (e.g., withdrawal of the umbilical catheter), open or percutaneous drainage of the abscess, and appropriate antibiotic treatment directed at the causative agent is expected to be curative. However, especially in developing countries, liver abscesses in preterm neonates are still associated with a high mortality rate of up to 50%, even with appropriate care and treatment [2]. The preterm neonate reported here had secondary liver abscess following TPN administration via an umbilical catheter and was treated successfully with broad spectrum antibiotics and surgical drainage of abscess.

## CASE REPORT

A premature newborn girl born at 33 weeks of gestation, weighing 2250 g was admitted to neonatal intensive care unit (NICU) with hydrops fetalis. Umbilical catheterization was done for IV access and for TPN. Nine days later, baby developed thrombocytopenia, leucocytosis, and abdominal distension. Blood and urine cultures were obtained, but no pathogen was detected. The umbilical catheter was withdrawn as it was thought to be a possible site of infection. Two days after catheter withdrawal, her clinical condition deteriorated. She was dehydrated and had abdominal distention, abdominal tenderness, and hepatomegaly. Bedside abdominal US revealed two compound cystic cavities, measuring 32×18 mm and 11×5 mm. US could not distinguish if these lesions were due to liver abscess, hematoma, or cavernous hemangioma. Laboratory analysis revealed WBC 13.8 103/µL, Hb 9.5 g/dL, PLT 32x103/µL, SGPT 79 U/L, SGOT 230 U/L, LDH 556 IU/L, and CRP 9.46 mg/L. Hemodynamic resuscitation were initiated. Repeat US the next day revealed a single, large, cystic cavity measuring 45×35 mm. Abdominal CT with intravenous contrast confirmed the diagnosis of liver abscess (Fig. 1). Vancomycin (15 mg/kg q6h IV) and meropenem (20 mg/kg q8h IV) were continued. After correction of her anemia, thrombocytopenia, and fluid and electrolyte disturbances, she underwent open surgical drainage of the abscess with lavage of the abscess cavity with warm saline and drainage with a suction drain. Culture of the pus from the abscess was negative. Postoperative recovery was uneventful, drain was removed on 5th postoperative day and baby was discharged on the 18th postoperative day. Regular follow-ups did not show any recurrence with normal ultrasound of liver US at 18 months.

**Figure:**
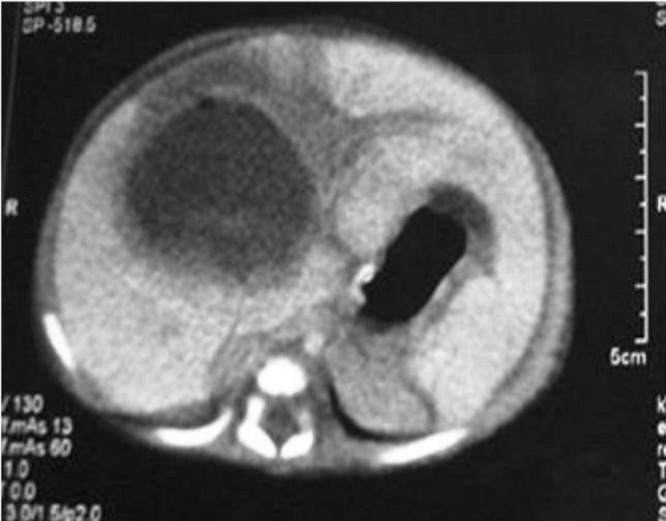
Figure 1: Abdominal CT with intravenous contrast revealed a single, large, cystic cavity measuring 45×35 mm. confirmed the diagnosis of liver abscess.

## DISCUSSION

Liver abscesses may be difficult to diagnose in neonates, partly due to their supposed rarity and lack of suspicion. Signs such as septic appearance, fever, intolerance to feeding, vomiting, abdominal distention, abdominal tenderness, and hepatomegaly are non-specific, as are laboratory findings such as leucocytosis, neutropenia, thrombocytopenia, increased ESR, and elevated or normal liver enzymes [3-5].

Incorrect placement of umbilical vein catheters and blood culture-proven sepsis are reported to be the most common predisposing factors for liver abscesses, followed by central TPN catheters, necrotizing enterocolitis, surgery, and prematurity with no other associated factors. We thought that the insertion of the umbilical catheter and prematurity were the causative factor for liver abscess in our case. Neutrophil defects have also been suggested to predispose neonates to liver abscesses [1,2]. The most common causative pathogens are Staphylococcus aureus, Streptococcus pyogenes, and E. Coli. Klebsiella, Pseudomonas, Corynebacterium acnes, anaerobes, and Candida albicans have also been cultured from neonatal liver abscesses. Polymicrobial infection is found in up to 50% of abscesses [1,6-8]. 

Treatment consists of correcting the predisposing factor (e.g., withdrawal of the umbilical catheter), percutaneous drainage with or with-out ultrasound guidance, or open drainage of the abscess affecting multiple lobes of the liver and/or large abscess as seen in our case. Appropriate antibiotic therapy should be directed to the causative agent [1,7]. In culture negative lesions, broad spectrum antibiotics covering both gram positive, gram negative organisms and anaerobes needs to be instituted. With prompt diagnosis and appropriate treatment, the outcome seems to be better as compared to a uniformly fatal state, though unfortunately, the mortality rate still can be as high as 50 % inspite of appropriate care and treatment [2].

In 2005, Tan et al reported 6 cases, and stated that there were fewer than 100 other reported cases in the English literature from 1930 to 2005 [1]. Since their report, additional 20 cases of liver abscesses in neonates have been reported, increasing the total number of recently reported cases to 26 [1,2, 4-6,9,10]. Bakshi et al. (2006) and Bari et al. (2007) included neonates in their reported case series of liver abscesses, but as they did not indicate the exact numbers of neonatal cases, those cases were not included in this report [7,11]. With our case, reported number of this rare condition amounts to 27 in last 6 years.

Over the past 6 years, the total number of reported neonatal liver abscess cases has increased by more than one-quarter since they were first reported in 1930. Availability of ultrasound especially the portable machine has been a major breakthrough to diagnose this condition and with improved awareness of this condition, there is an expected rise in the number of cases of neonatal liver abscess. 

In conclusion; performing routine bedside US to look for a liver abscess in all septic preterm or term newborns without an identifiable septic focus may ensure early diagnosis especially in those who are given TPN via umbilical catheter. Prompt and appropriate management is expected to improve the current outcome trends.

## Footnotes

**Source of Support:** Nil

**Conflict of Interest:**None
